# Validation of a CFD Model by Using 3D Sonic Anemometers to Analyse the Air Velocity Generated by an Air-Assisted Sprayer Equipped with Two Axial Fans

**DOI:** 10.3390/s150202399

**Published:** 2015-01-22

**Authors:** F. Javier García-Ramos, Hugo Malón, A. Javier Aguirre, Antonio Boné, Javier Puyuelo, Mariano Vidal

**Affiliations:** Superior Polytechnic School, University of Zaragoza, 22071 Huesca, Spain; E-Mails: hml@unizar.es (H.M.); javier.aguirre@unizar.es (A.J.A.); anbone@unizar.es (A.B.); javipuyuelo@gmail.com (J.P.); vidalcor@unizar.es (M.V.)

**Keywords:** air flow, air velocity, correlation, pesticide

## Abstract

A computational fluid dynamics (CFD) model of the air flow generated by an air-assisted sprayer equipped with two axial fans was developed and validated by practical experiments in the laboratory. The CFD model was developed by considering the total air flow supplied by the sprayer fan to be the main parameter, rather than the outlet air velocity. The model was developed for three air flows corresponding to three fan blade settings and assuming that the sprayer is stationary. Actual measurements of the air velocity near the sprayer were taken using 3D sonic anemometers. The workspace sprayer was divided into three sections, and the air velocity was measured in each section on both sides of the machine at a horizontal distance of 1.5, 2.5, and 3.5 m from the machine, and at heights of 1, 2, 3, and 4 m above the ground The coefficient of determination (R^2^) between the simulated and measured values was 0.859, which demonstrates a good correlation between the simulated and measured data. Considering the overall data, the air velocity values produced by the CFD model were not significantly different from the measured values.

## 1. Introduction

The air-assisted sprayers used in fruit production must be carefully and effectively regulated to ensure that crops are treated successfully. Four main factors affect the deposition efficiency: the nozzle type, the fluid pressure, the ground speed, and the volumetric flow rate of the air. The combination of these parameters determines the applied volume rate. This factor directly influences the quality of the treatment [1,2].

Considering the effect of the air volumetric flow rate, the fan setting directly affects the treatment quality [3]. If the air velocities or volumes produced by the fans are too low, insufficient pesticide will reach the trees. On the other hand, if these velocities or volumes are too high, the pesticide will be blown over and through the trees. Commonly, the operating manuals provided with these sprayers do not provide information on the characteristics of the air flow generated by the machine. In this sense, the air flow generated by the sprayer can be characterized by using high-precision anemometers such as sonic anemometers (2D and 3D) which are used to measure the velocity components for different heights, sections, and distances from the sprayer [4,5].

The use of experimental methods to characterize the air flow generated by a sprayer would be difficult and expensive [6]. Instead, the air velocity in the vicinity of a sprayer can be simulated by applying integrated computational fluid dynamics (CFD) [7,8]. CFD models are a useful tool for improving the design and development of these machines by enabling the testing of different design alternatives and reducing the number of measurements to those required to validate the models. Nevertheless, some measured data remains necessary and, to achieve this, the use of high-precision anemometers is required.

CFD models can be used to estimate the air velocity by considering the sprayer to be either stationary or moving [9–11]. The first step in analysing the air flow generated by a sprayer consists of determining the air flow patterns while the sprayer is stationary, with no forward motion of the sprayer and without considering any interaction with the crop [11]. The static characterisation of the air flow pattern allows us to understand how the sprayer will perform when moving forward at a certain speed. Several studies have shown the relationship between the measurements obtained when the sprayer is either stationary or moving forward [10,11], whereby the air flow pattern of a moving sprayer exhibits a decrease in the air velocity relative to the values obtained when the sprayer is stationary [10]. This decrease becomes more pronounced as the forward speed of the sprayer increases.

CFD models have been successfully applied to the simulation of the operation of different sprayers used in agriculture: boom sprayers [12], the rotary atomizers used on agricultural aircraft for ultra-low volume spraying operations [13], recycling tunnel sprayers for orchards [14], and to a greater degree, in the air-assisted sprayers used in orchards [15,16].

Although 2D CFD models were used originally, 3D models are better suited to representing the air flow generated by a sprayer [17]. In fact, CFD models have been improved to study further aspects affecting the efficacy of the treatment, including the interaction between the air flow and the crop or the influence of environmental parameters (wind speed, temperature) on the treatment [18,19].

Most CFD models that have been developed to simulate the air flow generated by an air-assisted sprayer have assumed the use of the air velocity measured at the fan outlet as a boundary condition [6,11,20]. This methodology is very useful for analysing the characteristics of the air flow generated by a sprayer considering its specific configuration: outlet width, deflector position, fan blade angle, and fan speed. However, taking the design of the sprayer into consideration, it is important to obtain simulation models capable of simulating the air flow produced by the fan at different settings, so that the air velocity at the fan outlet is not a boundary condition. This type of model would be a flexible tool for simulating different air outlet geometries for a specific air flow, thus avoiding the need to measure the air speed at the fan outlet for each design. In this case, the total air flow produced by the sprayer must be measured for each fan configuration, requiring experimental work that, in any case, the sprayer manufacturer must perform to obtain technical information on the air flow generated by the fan. This is necessary because the operational adjustment of a sprayer should be based on a knowledge of the air flow created by the fan and the geometry of the discharge outlet [10].

The air flow pattern depends on the number and configuration of the sprayer fans. Currently, conventional orchard sprayers consist of a fan located behind a tank. For this kind of machine, different studies have shown the viability of using CFD to estimate the generated air velocity [6,11,15,16,18–20]. However, no CFD models have been developed for a sprayer equipped with two axial fans. This kind of machine has been developed in recent years, with several different designs appearing [7,21]: two fans placed side by side behind the tank, two fans placed in line behind the tank, one fan placed in front of the tank and another behind, two fans behind the tank at different heights, and so on. In addition, the models have been based on the use of air velocities measured at the fan outlet as a boundary condition.

The objective of this work was to develop and validate, by experiment, a CFD model for predicting the air velocity distributions for an air-assisted sprayer equipped with two axial fans, assuming the total air flow instead of the outlet air velocity to be the main parameter of the CFD model and to validate the model by using 3D sonic anemometers to take actual measurements. The model was developed for different fan speeds and assuming the sprayer to be stationary.

## 2. Experimental Section

### 2.1. Air Sprayer

**Figure 1.**
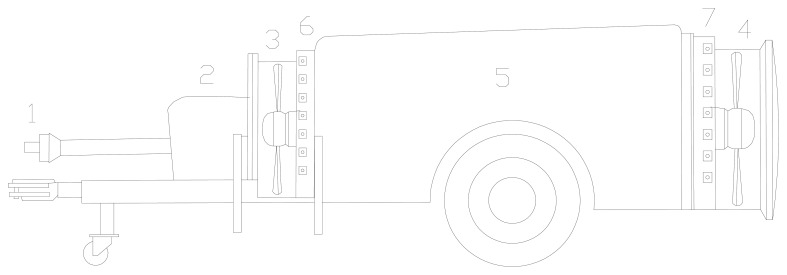
Air-assisted sprayer equipped with two reversed-rotation axial fans. 1. PTO; 2. Pump; 3. Front fan; 4. Rear fan; 5. Tank; 6. Front nozzles; 7. Rear nozzles.

**Figure 2.**
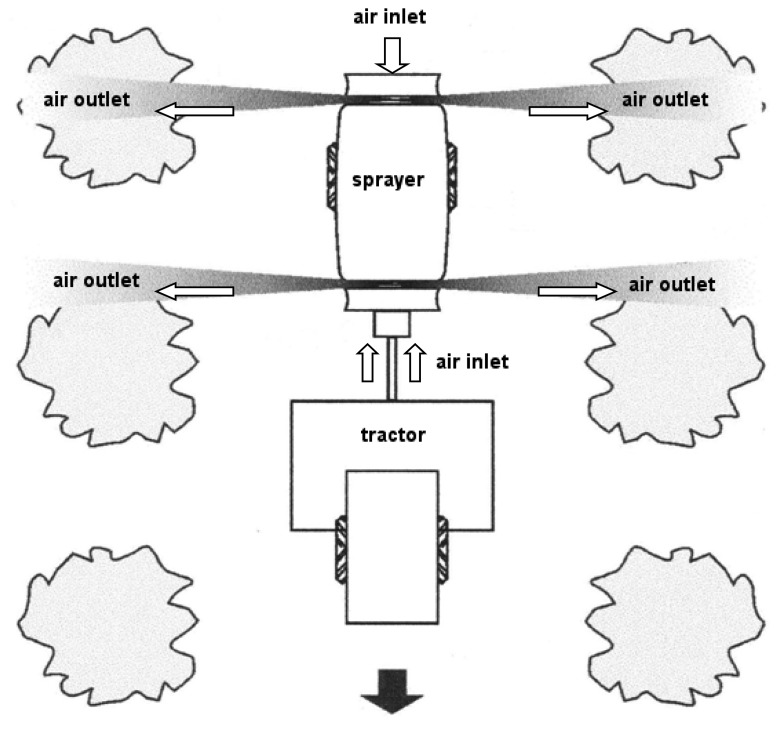
Air flow produced by the front and back fans.

The analysed sprayer was a product of Gar Melet S. L. (Huesca, Spain). The sprayer (Figure 1) was equipped with two reversed-rotation axial fans [4], one placed behind the tank and the other placed in front. These fans spin in opposite directions to ensure a uniform distribution of the chemical product to be applied. When viewed from the tractor the front fan spins anticlockwise and the rear fan clockwise. Each fan sucks air axially from the outer area of the machine and throws it radially (Figure 2). The diameter of the front fan was 800 mm and that of the rear fan was 830 mm. The CFD model was developed assuming the operation of both of these fans.

The fan blade angles can be adjusted, through a range enumerated as 1 to 5, to create different air flows. The air flows were measured according to the ISO 9898:2000 standard [22], considering three fan blade positions (1.5; 3; 4.5). The air flow produced by the rear fan was measured at the fan's inlet, using a TESTO 0635 1041 hot-wire anemometer (Testo AG, Lenzkirch, Germany; accuracy: 0.03 m/s; range: 0 to 20 m/s). The measurements were carried out with the power take-off (PTO) turning at 540 rpm.

### 2.2. Experimental Measurements

The velocity of the air generated by the sprayer was measured in the absence of any wind by using a WindMaster 3D sonic anemometer (Gill Instruments, Lymington, UK) according to the methodology developed by García-Ramos *et al.* [4]. The accuracy of the sonic anemometer was 1.5% (for wind speeds up to the maximum measureable value) with an air velocity range of 0 to 45 m/s, and a resolution of 0.01 m/s. The air velocity data was recorded at a frequency of 1 Hz.

**Figure 3.**
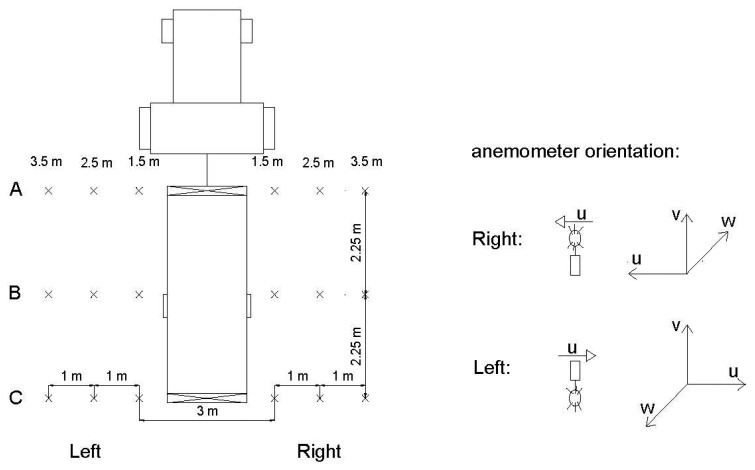
Measuring points and anemometer orientation used for static analysis of the sprayer operating and stationary.

**Figure 4.**
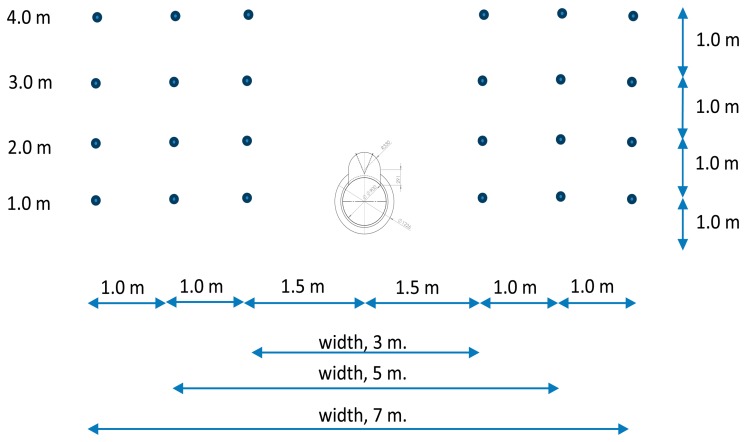
Air velocity measurement points for each section of the sprayer.

Measurements were carried out with the sprayer operating but stationary, for three different settings of the rear fan blades (1.5, 3, and 4.5) and, therefore, three different air volumetric flow rates (Table 1). The air velocity was measured in three sections of the sprayer: A, B, and C (Figure 3). For each section, measurements were performed on both sides of the machine at 1.5, 2.5, and 3.5 m from the centre of the sprayer, at heights of 1, 2, 3, and 4 m above the ground (Figure 4). For each measuring point and sprayer setting, the air velocity was recorded over a 60-s duration. The anemometer orientation for the measurements is shown in Figure 3.

### 2.3. Model Formulation

A CFD simulation software, specifically, the commercial ANSYS-CFX 14.5 CFD code, was used to analyse the air flow generated by the sprayer. This study used the Spalart–Allmaras model [23]. This is a single-equation model (Equation (1)) that solves a modelled transport equation for a turbulent kinematic viscosity. The Spalart–Allmaras model is implemented specifically for applications involving wall-bound flows [24–26]. For this reason, this model is used for turbomachinery applications [27–30]. The Spalart–Allmaras model adds an extra condition, specifically, that turbulent kinematic viscosity at the walls is zero:

(1) $$\frac{\partial \bar{v}}{\partial t}+u_{i}\frac{\partial \bar{v}}{\partial x_{i}}=G_{v}+\frac{1}{\sigma }\left[\nabla \left(v+\bar{v}\right)\;\nabla \bar{v}\right]+c_{b2}{\left|\nabla v\right|}^{2}-Y_{v}$$

(2) $$G_{v}=c_{b1}\left[1-f_{t2}\right]\;\tilde{S}\bar{v}+\frac{c_{b1}}{k^{2}}f_{t2}{\left(\frac{\bar{v}}{d}\right)}^{2}$$

(3) $$Y_{v}=c_{w1}f_{w}{\left(\frac{\bar{v}}{d}\right)}^{2}$$

Equation (1) represents the governing equation for the Spalart–Allmaras model. In these equations, *G_v_* and *Y_v_* are the production and the destruction of turbulent viscosity, respectively (Equations (2) and (3)). This effect occurs in the near-wall region due to the effects of wall blocking and viscous damping.

In Equation (1), *u* represents the fluid velocity (obtained previously by solving the Navier–Stokes (N–S) equations), *v* is the molecular kinematic viscosity, and *d* (Equations (2) and (3)) is the distance to the closest surface, with:

$$\begin{array}{c} \begin{array}{cccc} \tilde{S}=S+\frac{\bar{v}}{k^{2}d^{2}}f_{v2}; & f_{v2}=1-\frac{X}{1+Xf_{v1}}; & f_{v1}=\frac{X^{3}}{X^{3}+{C_{v1}}^{3}}; & C_{v1}=7.1 \end{array}\\ \begin{array}{cc} S=\sqrt{2\Omega_{ij}\Omega_{ij}}; & \Omega_{ij}=\frac{1}{2}\left(\frac{\partial u_{i}}{\partial x_{j}}-\frac{\partial u_{j}}{\partial x_{i}}\right) \end{array}\\ \begin{array}{cccc} f_{w}=g{\left[\frac{1+{C_{w3}}^{6}}{g+{C_{w3}}^{6}}\right]}^{1/6}; & g=r+C_{w2}(r^{6}-r); & r=\frac{\bar{v}}{\tilde{S}k^{2}d^{2}}; & C_{w2}=0.3 \end{array}\\ \begin{array}{ccc} f_{t2}=C_{t3}\exp(-C_{t4}X^{2}); & C_{t3}=1.2; & C_{t4}=0.5 \end{array}\\ \begin{array}{ccccc} C_{w1}=\frac{C_{b1}}{k^{2}}+\frac{\left(1+C_{b2}\right)}{\sigma }; & C_{b1}=0.1355; & C_{b2}=0.622; & K=0.41; & \sigma =\frac{2}{3} \end{array} \end{array}$$

The turbulent kinematic viscosity, *v_t_*, is given by:

$$\begin{array}{ccc} v_{t}=\bar{v}f_{v1}; & f_{v1}=\frac{X^{3}}{X^{3}+{C_{v1}}^{3}}; & X=\frac{\bar{v}}{v} \end{array}$$

Detailed information about the Spalart-Allmaras model is given in [23].

### 2.4. Simulation Domain and Load Cases

**Figure 5.**
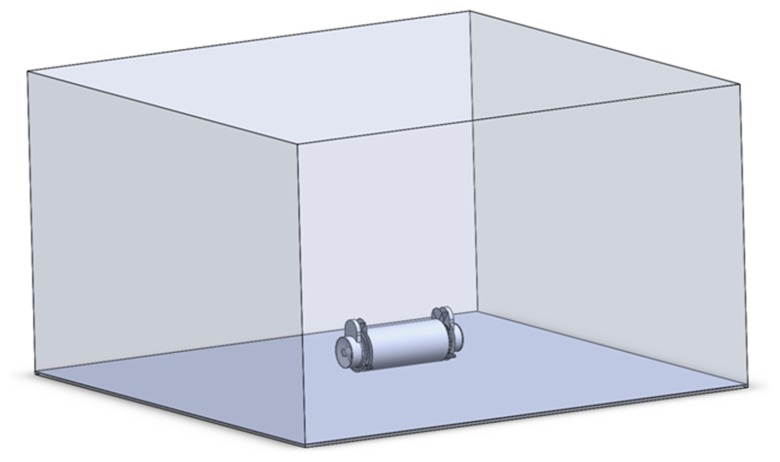
CFD simulation domain for numerical analysis of the air flow generated by the sprayer.

To develop the study, a numerical analysis was performed. The simulation domain used in the numerical analysis was 15 m long in the driving direction, 15 m wide, and 8.9 m high. These dimensions are necessary to avoid errors in the results due to the contours of the simulation domain. Figure 5 shows the simulation domain.

**Figure 6.**
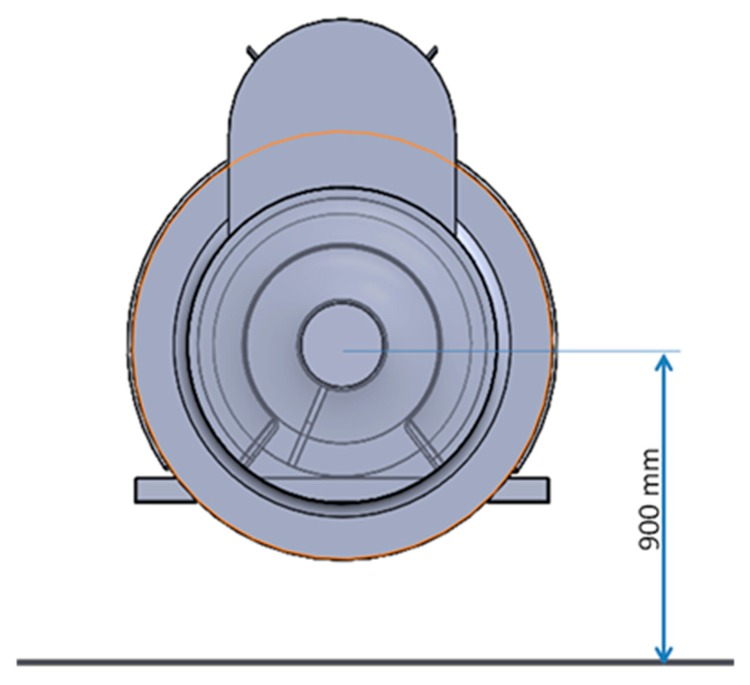
Location of rotors of the fans of the sprayer in the CFD simulation domain.

In this simulation domain, the research group modelled an air-assisted sprayer with two fans. The air-assisted sprayer was located in the centre of the domain base, with the rotors of the fans 0.9 m above ground level, as shown in Figure 6. The diameters of the front and rear fans were 0.8 m and 0.9 m, respectively. The distance between the fans was 3 m. In addition, the air-assisted sprayer was equipped with a water tank between the two fans, which was also modelled.

The tractor was assumed to be stationary during the research carried out. Considering the air flow at the entrance of the fans, the actual air flows were measured experimentally in such way that the effect of the tractor on the air input was included in the measurement. On the other hand, the CFD numerical model considered the experimental air flow measured at the entrance of both fans as input parameter, then, the air flow sucked by the fans in the numerical analysis was not affected by the presence of the tractor because it was an input parameter. Considering the air velocity generated by each fan in the vicinity of the sprayer, the air flows generated in static way were perpendicular to the tractor-sprayer system (Figure 2). In this sense, the tractor does not affect the air velocity values at the measurement points for each section of the sprayer. For this reasons, the effects of the tractor on the results would be negligible. Consequently, the tractor was not included in the numerical model.

The meshing process was carried out in two phases. In the first phase, an optimizing mesh process was carried out in order to fulfill the convergence criteria imposed. The convergence criterion applied in the study was that residual values obtained in the numerical analysis were below 10^−4^. Once the convergence criterion was fulfilled, a mesh-independence study was carried out in order to verify the accuracy of the numerical model. In this study the number of elements has been increased up to 50% with respect to the initial number of elements. The numerical results obtained from the different meshes generated in the mesh-independence study showed similar values, with mean differences around 2.1%, but the computational cost was greatly increased by increasing the number of elements in the model. Once this process was finished, the conclusion obtained was that the numerical model obtained after the optimization phase of the mesh was correct. This numerical model was configured using hexahedral and shell elements. The hexahedral elements were used to simulate the fluid, while the shell elements were used to define the fans of the air-assisted sprayer and the sides of the simulation domain. The model consisted of 4,682,881 nodes and 948,735 elements.

The first boundary condition imposed in the simulation was to constrain the movement of the air-assisted sprayer. In addition, the air flow generated by the fans can cross the lateral sides and top of the simulation domain. In these areas a pressure outlet boundary condition has been imposed. At the bottom of the domain, the air flow cannot cross the ground.

In the developed study, three load cases were analysed. These corresponded to three possible settings of the fan blades. The manufacturer of the sprayer enumerates these positions as “1.5, 3, and 4.5”.

**Figure 7.**
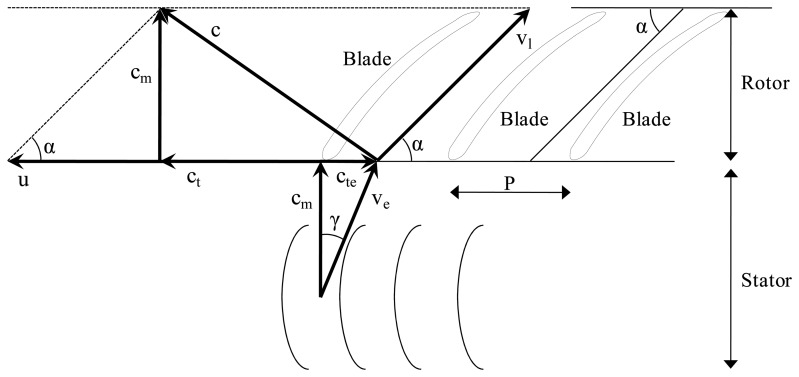
Axial (c_m_) and rotational (c_t_) velocity of the air at the outlet of the fan blades of the sprayer fan. γ: inclination of the stator blades; α: inclination of the rotor blades; v_l_: linear velocity of the blades; c: output air speed of the fan; v_e_: inlet air speed at the rotor; P: distance between the blades of the rotor.

To implement the simulation, two components of the air velocity in the fan are required for each load case. These components are the axial (c_m_) and rotational (c_t_) velocity of the air at the outlet of the fan blades (Figure 7). The values of c_m_ and c_t_ were calculated using the equations derived from Figure 7.

In modern air-assisted sprayers, the fans have two types of blades. The first of these are fixed blades, called “stators”, while the other type can move, and are called “rotors”. The function of the stators is to guide the air flow so as to improve the performance of the machine when the air comes into contact with the moving blades. The inclinations of the stator blades and rotor blades are designated γ and α, respectively.

The initial data for the analysis, in addition to the inclinations of the blades, includes the geometric data of the machine as well as the air flow to the fan inlet (Q, Table 1) and the angular velocity of the blades (w). The values of Q and w were obtained by experiment.

The c_te_ parameter corresponds to the rotational component of the air flow caused by the stator. This is calculated from the axial component of the air flow at the fan inlet (c_m_) and the inclination of the stator blades (γ).

The c_t_ parameter corresponds to the rotational component of the air flow caused by the relative velocity of the rotor. It is calculated from the axial component of the air flow at the fan inlet (c_m_) and the inclination of the rotor blades (α).

Equations (4)–(8) show the relationship between the parameters shown in Figure 6.

(4) $$\vec{\text{Q}}=\text{S}\cdot \vec{{\text{c}}_{\text{m}}}$$

(5) $$\vec{{\text{v}}_{\text{e}}}=\vec{{\text{c}}_{\text{te}}}+\vec{{\text{c}}_{\text{m}}}$$

(6) $$\vec{\text{c}}=\vec{\text{u}}+{\vec{\text{v}}}_{\text{l}}$$

(7) $$\vec{\text{c}}=\vec{{\text{c}}_{\text{m}}}+\vec{{\text{c}}_{\text{t}}}$$

(8) $$\vec{\text{u}}=\text{R}\cdot \vec{\text{w}}$$

The values of the parameters used in this study are listed in Table 1, where S and r are the air passage of the fan and the radius of the fan, respectively.

### 2.5. Statistical Analysis

The development of the CFD model was analysed by calculating the coefficient of determination (R^2^) between the simulated and actual measurements. To analyze the effect of distance to the machine (horizontal and vertical) we calculated the correlation between the two measures, assuming that both fans are running, for all possible configurations.

The effects of the main variables affecting the air velocity values, as determined with the CFD model (Table 2), were analysed statistically. A generalized linear model with a gamma distribution and a logarithmic link function was used to establish the principal effect of the variables on the air velocity [31]. In addition, two-way interactions between the variable “analysis type” and other fixed effects were analysed. The analysis of other interactions was not considered relevant to validate the viability of the CFD model.

A total of 432 data were used in the statistical analysis, 216 obtained from the experimental measurements (considering the mean air velocity for each measurement point), and 216 from the CFD model (Table 2).

To analyse the differences between the measured and simulated values, the data matrix was partitioned and the data was analysed, one on one, for each combination of variables, paying attention to the significance of the double interactions after completion of the generalized linear model. The set of experimental observations of the variable velocity of the air generated by the sprayer follows a normal distribution and homocedasticity, however, obtained with CFD model does not (Kolomogorov-Smirnov test 0.226; *p* < 0.001). The air velocity was transformed into a logarithmic function to obtain a normalized distribution. The Student t test was used, with an appropriate value being chosen according to the homogeneity of the variances after the application of the Levene test. All statistical analyzes were performed with SAS (SAS Inst. Inc., Cary, NC, USA).

## 3. Results and Discussion

### 3.1. Correlation between CFD and Measured Results

**Figure 8.**
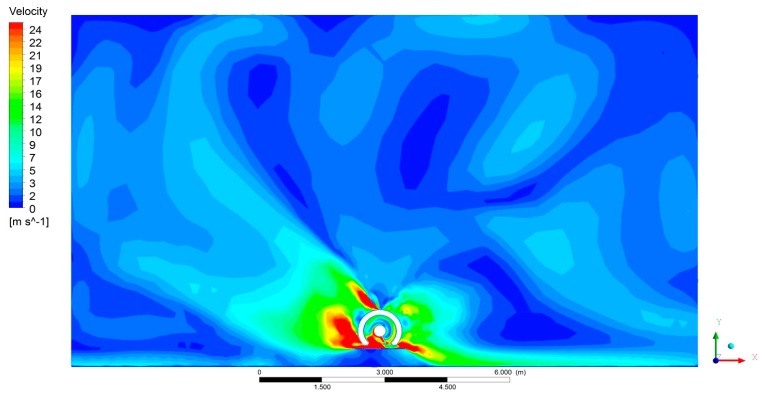
Air velocity obtained with CFD model in front section of sprayer (A). Both the front and rear fans are running. The fans are set to position 3.

**Figure 9.**
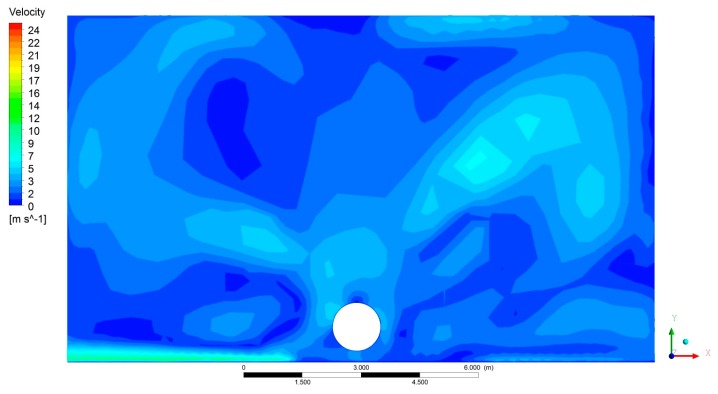
Air velocity obtained with CFD model in centre section of sprayer (B). Both the front and rear fans are activated. The fans are set to position 3.

**Figure 10.**
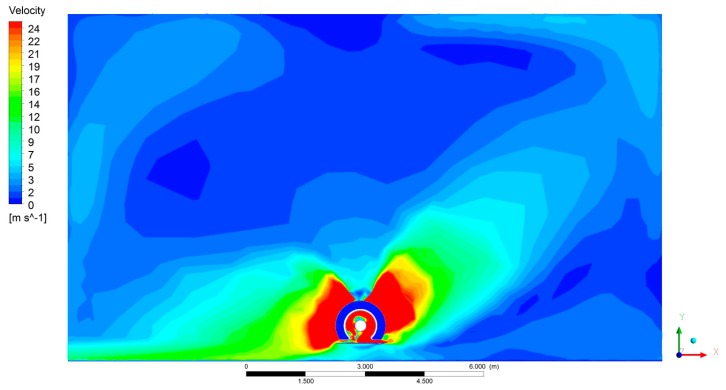
Air velocity obtained with CFD model in rear section of sprayer (C). Both the front and rear fans are activated. The fans are set to position 3.

Figures 8, 9 and 10 show the CFD results for a specific fan configuration (both front and rear fans set to fan setting 3), considering three sections of the sprayer and viewing the sprayer from behind. The simulation shows the asymmetry of the air flow generated by the fans due to the direction in which it is turning. Because of this, the use of two fans spinning in opposite directions compensates for the effect of the asymmetry, thus producing an overall air flow that is more uniform on both sides of the machine.

The air velocity values generated by the CFD model were in good agreement with those obtained by [11] who showed, through a test with a stationary traditional sprayer, that the air velocities decreased with the distance and height from the sprayer, but increased with the air flow. The variation in the air velocity values was similar to that obtained by [10], who concluded that, for a static test using a traditional sprayer, the air velocities at a distance of 3.5 m were half the value at 1.75 m.

The overall R and R^2^ coefficients, considering all of the data, were 0.927 and 0.859, respectively. This shows that there is a good correlation between the simulated and measured data. The results were excellent (Table 3), with R^2^ more than 0.856, with an improvement in the areas closest to the sprayer.

Considering the effect of the air flow (fan settings of 1.5, 3, and 4.5) the best adjustment was obtained at the lower air flows. On the other hand, the worst adjustment was that corresponding to a fan setting of 4.5, although the correlations were satisfactory with R^2^ = 0.825.

The obtained correlation values indicate that the CFD model can be used as a useful tool for predicting the actual values of the air velocity in the vicinity of a sprayer. However, it is necessary to analyse the error level and the influence of each variable (distance, height, air flow) in the model in more detail.

### 3.2. Influence of Spraying Variables on CFD Model

The air velocity was significantly affected by all of the variables except for the “analysis type” and the “sprayer side” (Table 4). The fact that the “analysis type” had no significant effect on the air velocity, coupled with the high values of the coefficients of determination, supports the validity of the use of the CFD model to estimate the air velocity in the vicinity of the sprayer.

Considering the effect of the “sprayer side” on the CFD and measured air velocities, no significant differences were found between the different sides of the sprayer. This fact has been analysed by other researchers [10], who concluded that, for most sprayers, it is possible to observe at least a small difference in the air field between the left and right sides. On the other hand, a sprayer design in which the fans turn in opposite directions produces an air flow that is symmetrically distributed.

The degree of influence of the variables in the CFD model on the air velocity, are listed in Table 4. In descending order of importance, they are measurement section, height, horizontal distance, air flow, analysis type, and sprayer side.

Considering the interaction of the “analysis type” with the other variables, it was not significant for “air flow”, “sprayer side” and “height”. However, two significant interactions were found for the case of “analysis type” × “measurement section” and “analysis type” × “horizontal distance” (Table 4).

**Figure 11.**
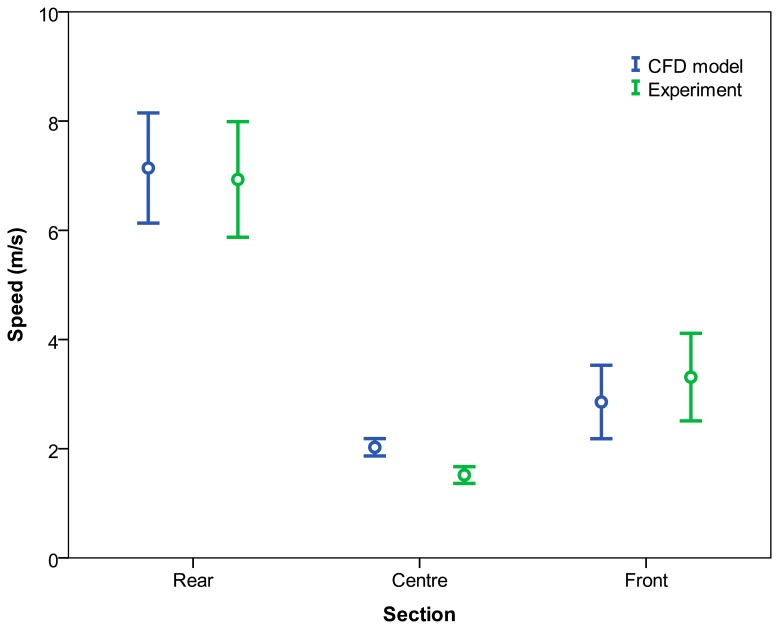
95% confidence interval of means of air velocity values according to analysis type and measurement section.

Considering the effect of the “measurement section”, the velocity values were found to be significantly higher in the centre section for the CFD model. However, no significant differences were found in the front and rear sections (Table 5). Figure 11 shows the 95% confidence interval. Considering the mean values for the air velocity listed in Table 5, the mean absolute errors obtained were 3.79 for the rear section, 33.55% for the centre section, and 13.89% for the front section.

This fact is concordant with that shown in Table 4 which reflects a significant effect of the interaction of the variables “analysis type” and “measurement section” on the air velocity values. Considering the interaction between the variables, this significant effect (Table 4) is explained because the CFD data were lower than experimental ones for the front section and, by the other hand, for rear and centre sections, the CFD model values were higher than the experimental ones (Table 5).

**Figure 12.**
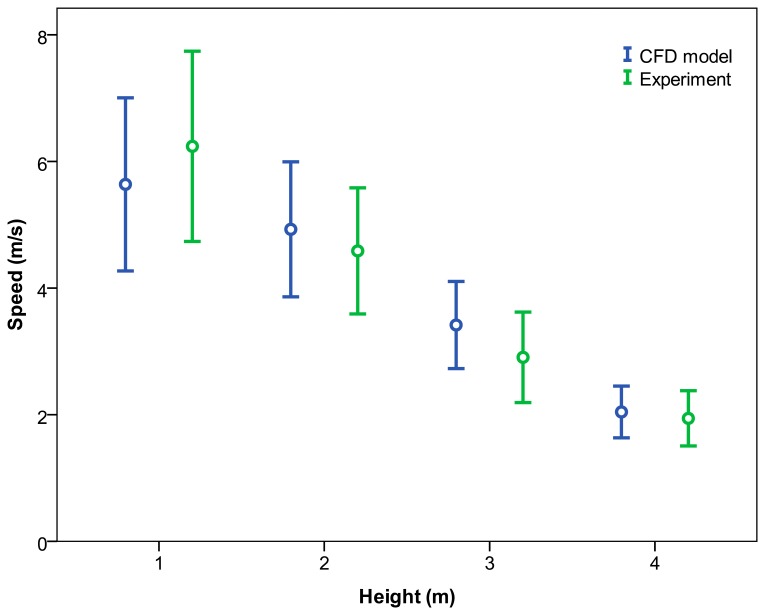
95% confidence interval of means of air velocity values according to analysis type and height.

For the variable “height”, no significant differences were obtained except at a height of 3 m (Table 6 and Figure 12). The air velocities were found to fall with an increase in the measurement height. Considering that Mediterranean fruit orchards are typically between 1 and 4 m in height, the CFD model was able to predict the air velocities with a mean error of 9.92%.

**Figure 13.**
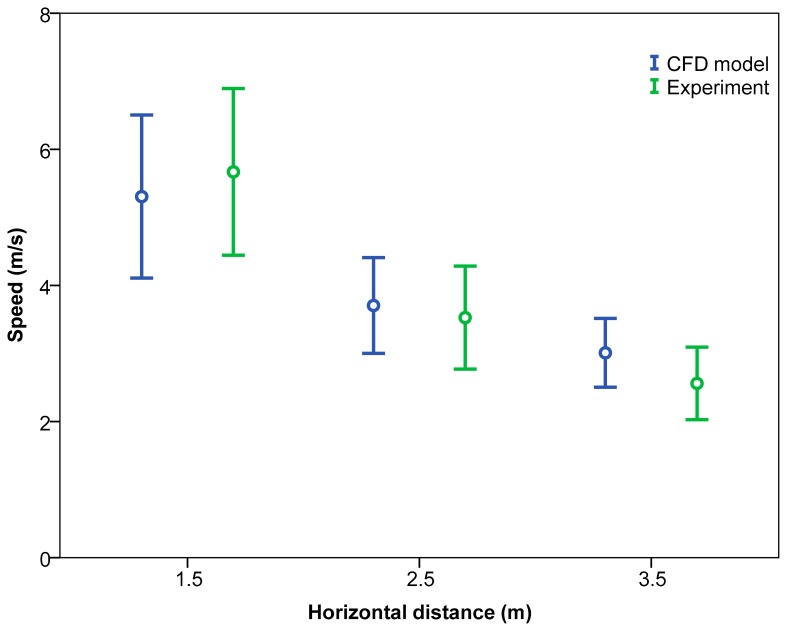
95% confidence interval of means of air velocity values according to analysis type and horizontal distance.

The air velocity values obtained with the CFD model did not differ significantly from the measured values for horizontal distances up to 2.5 m (Table 7 and Figure 13) with a mean error of 5.72%. This means that, given that fruit orchards typically have planting widths of 5 m, the CFD model is in fairly good agreement with the measured values. This fact is concordant with that shown in Table 4 which reflects a significant effect of the interaction of the variables “analysis type” and “horizontal distance” on the air velocity values, showing that. Analysing this interaction, and considering low horizontal distances (until 1.5 m), CFD data were lower than experimental ones However this fact was the opposite for distances of 2.5 and 3.5 m where the CFD model values were higher than the experimental ones.

**Figure 14.**
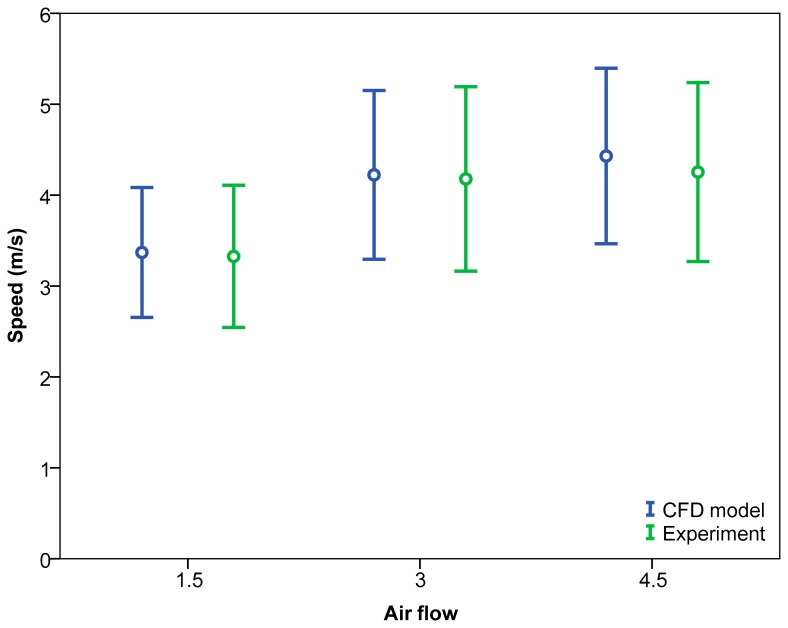
95% confidence interval of means of air velocity values according to analysis type and air flow generated by fans.

Upon analysing the effects of the air flow generated by the fans on the air velocity values (Table 8 and Figure 14), no significant differences were found. The results were similar for both the low and high air flows with no clear trends apparent.

A comparison of the measured and estimated values for the air velocity, considering a zone no more than 3 m in height and less than 2.5 m from the sprayer, which corresponds to a typical treatment area in a Mediterranean fruit orchard, revealed a mean error of 9.19%. These results were in good agreement with those of previous research. In this sense, [11] incurred error of 25% for a sprayer equipped with a rear fan and operating while stationary, at a measurement distance of 1.75 m. In a similar way, [19] incurred errors of less than 20% in 95% of the measurements made for three sprayers at forward speeds of 7.1 km/h, with mean values having an error of less than 11%.

### 3.3. Usefulness of the CFD Model

The obtained correlation values between experimental and simulated data indicate that the CFD model can be used as a useful tool for predicting the actual values of the air velocity in the vicinity of the sprayer. Besides, information of Table 4 demonstrates the usefulness of the CFD model since the statistical analysis carried out shows that the results obtained by numerical simulation were not significantly different from those obtained experimentally. This fact, added to the high values of the coefficient of determinations between experiment and model (Table 3), supports the use of the CFD model as a valid alternative to the experimental methods. In addition, the CFD model has shown its robustness to analyze the relative importance of the variables that affect the characteristics of the airflow generated as it has been showed in Tables 5, 6, 7 and 8. The fitting of the data supplied by the CFD model with the experimental measurements also supports the use of the Spalart Allmaras turbulence model to analyse the air flow generated by the sprayer.

Considering the experimental measurements, the research shows the utility of using high precision sensors (sonic and hot-wire anemometers) to measure the components of the air velocity for different heights, sections, and distances from the sprayer. The use of this type of sensors is required to obtain accurate experimental data which will be used to validate the CFD model. This fact is in concordance with previous studies [4,5].

As conclusion, one of the main advantages of the proposal numerical model is the use, for each fan, of the total air flow aspirated as the main parameter to be introduced in the model instead of the outlet air velocities used in traditional models. This methodology reduces the requirement of specific experimental measurements for each configuration of air flow in the fans, and reduces the time required to analyse the performance of a sprayer equipped with two fans. In this sense, the proposed CFD model will be an useful tool for sprayer manufacturers to improve the design phase of the machine by predicting, for different fan configurations, the characteristics of the air velocities generated by the sprayer in the vicinity of the machine which will let to analyse the influence of different designs of the machine, with different dispositions of the front and back fans.

## 4. Conclusions

The application of CFD models to the estimation of the air velocity distributions generated by an air-assisted sprayer equipped with two axial fans and operating while stationary, considering the total air flow rather than the outlet air velocity as the main parameter of the CFD model, was validated as being an effective method.

Considering all of the data, the air velocity values obtained with the CFD model were found to be in good agreement with the measured data. The global coefficient of determination between the CFD model and measured data was 0.859. Considering a zone of up to 3 m in height and 2.5 m from the sprayer, the mean error between the measured and estimated values was found to be 9.19%. The degree of influence of the variables used in the CFD model on the air velocity, in descending order of importance, was: the measurement section, height, horizontal distance, air flow, analysis type, and sprayer side. Significant differences arose only for specific combinations of three variables, with the values predicted by the computer simulation being significantly higher than those measured by experiment. These variables were, in descending order of importance, the measurement section (centre), height (3 m), and horizontal distance (3.5 m).

## Figures and Tables

**Table 1.** Input data for CFD analysis.

**Parameter**	**Rear Fan Air Flow**			**Front Fan Air Flow**		
		
**Fan Setting**	**1.5**	**3**	**4.5**	**1.5**	**3**	**4.5**
**c_te_ (m/s)**	13.03	17.56	19.38			
**γ(°)**	60	60	60	60	60	60
**W (rad/s)**	255	255	237	198	198	184
**α (°)**	36.91	47.16	58.21	36.91	47.16	58.21
**u (m/s)**	76.5	76.5	71.1	59.3	59.3	55.2
**S (m^2^)**	0.59	0.59	0.59	0.46	0.46	0.46
**v_e_ (m/s)**	26.06	35.12	38.76			
**c_m_ (m/s)**	22.57	30.42	33.57	16.62	23.51	26.76
**c_t_ (m/s)**	33.40	30.71	30.90	37.18	37.49	38.60
**Q (m^3^/s)**	13.31	17.94	19.8	7.60	10.81	12.32
**r (m)**	0.45	0.45	0.45	0.4	0.4	0.4
**w_s_ (rad/s)**	111	102	103	124	125	128

**Table 2.** Variables affecting air velocity values determined with CFD model. N: number of observations.

**Variable Name**	**Variable Configurations**	**N (Each Level)**
Analysis type	Experimental; simulated (CFD)	216
Measurement section	Front (A); Centre (B); Rear (C)	72
Air flow	1.5; 3; 4.5	24
Sprayer side (viewed from behind)	Left; Right	12
Horizontal distance	1.5 m; 2.5 m; 3.5 m	4
Height	1 m; 2 m; 3 m; 4 m	1

**Table 3.** Coefficients of determination (R^2^) according to horizontal and vertical distance from machine. Overall values for three measurement sections (A, B, C) and three fan settings (1.5, 3, 4.5).

**Height (m)**	**Horizontal Distance (m)**		

	**3**	**5**	**7**
**4**	0.911	0.883	0.856
**3**	0.922	0.885	0.895
**2**	0.971	0.947	0.929

**Table 4.** Effect of variables on air velocity when using generalized linear model with sum of squares Type III, results contrast Omnibus: Chi square likelihood ratio = 440.292; *p* < 0.001. Deviation obtained with this model in relation to a gamma distribution with a logarithmic link function was 137.738.

**Variable (Interactions)**		**Wald Chi-Square**	**df**	**Significance**
**(Interception)**		1806.389	1	<0.001
**Analysis type**		3.054	1	0.081
**Measurement section**		368.217	2	<0.001
**Air flow**		15.722	2	<0.001
**Sprayer side**		0.498	1	0.480
**Horizontal distance**		79.843	2	<0.001
**Height**		137.583	3	<0.001

**Analysis type ×**	**Measurement section**	14.455	2	<0.001
	**Air flow**	0.670	2	0.715
	**Sprayer side**	0.801	1	0.371
	**Horizontal distance**	13.388	2	<0.001
	**Height**	6.691	3	0.082

**Table 5.** Air velocity values according to analysis type and measurement section. The Student t test was used to compare the means.

**Measurement Section**	**Analysis Type**		**Probability**			
	
	**CFD**	**Measured**	**SEM**	**t**	**p**	
**Rear**	6.83	6.58	0.25	0.374	0.709
**Centre**	2.03	1.52	0.51	4.586	<0.001
**Front**	2.85	3.31	0.45	−0.311	0.756

**Table 6.** Air velocity values according to the analysis type and the height. The Student t test was used to compare the means.

**Height (m)**	**Analysis Type**		**Probability**			
	
	**CFD**	**Measured**	**SEM**	**t**	**p**
1	5.64	6.24	0.60	−0.593	0.555	
2	4.93	4.59	0.34	0.468	0.640	
3	3.42	2.91	0.51	2.006	0.048	
4	2.04	1.94	0.10	0.716	0.475	

**Table 7.** Air velocity values according to analysis type and horizontal distance. The Student t test was used to compare the means.

**Horizontal Distance (m)**	**Analysis Type**		**Probability**		
	
	**CFD**	**Measured**	**SEM**	**t**	**p**
1.5	5.31	5.67	0.36	−0.425	0.672
2.5	3.71	3.53	0.18	0.966	0.336
3.5	3.01	2.56	0.45	2.062	0.041

**Table 8.** Air velocity values of air flows generated by fans. The Student t test was used to compare the means.

**Air Flow**	**Analysis Type**		**Probability**		
	
	**CFD**	**Measured**	**SEM**	**t**	**p**
1.5	3.37	3.33	0.04	0.637	0.525
3	4.22	4.18	0.04	0.800	0.425
4.5	4.43	4.25	0.17	0.735	0.464
